# Western Models of PTSD Rehabilitation Among Military Veterans: A Narrative Comparative Review and Policy Implications for Israel

**DOI:** 10.3390/healthcare14070929

**Published:** 2026-04-02

**Authors:** Dotan Braun, Maya Lusky, Yoram Ben Yehuda, Eyal Fruchter

**Affiliations:** 1 Trauma and PTSD Clinic, Rishon LeZion 7559104, Israel; 2 ICAR Collective, Modiin 7177908, Israelfrueyal@gmail.com (E.F.); 3 Ways Institute, Rehovot 76606, Israel; 4 Dina Recanati School of Medicine, Reichman University, Herzliya 4610101, Israel

**Keywords:** PTSD, veterans, rehabilitation, stigma, peer support, biopsychosocial-spiritual, Israel, health policy

## Abstract

**Background:** Post-Traumatic Stress Disorder (PTSD) is among the most prevalent and disabling mental health conditions affecting military veterans in Western countries. In recent decades, PTSD has increasingly been conceptualized as a systemic neuropsychological injury shaped not only by individual psychopathology, but also by institutional, cultural, and political contexts, particularly in settings of prolonged conflict and political violence. This shift has given rise to diverse national rehabilitation models that extend beyond symptom-focused care. This narrative comparative review aims to examine national models of PTSD rehabilitation among military veterans and to derive policy-relevant insights for Israel. **Methods:** We conducted a narrative comparative review of peer-reviewed literature and national policy documents published between 2014 and 2023, examining military and veteran PTSD rehabilitation frameworks in six Western countries: the United States, Canada, the United Kingdom, Germany, Australia, and the Netherlands. Sources were identified through PubMed, PsycINFO, Google Scholar, and governmental repositories. The review focused on system-level rehabilitation structures, including clinical services, peer-based programs, occupational integration, community and cultural components, and national monitoring practices. **Results:** Across countries, recurring challenges included persistent stigma limiting help-seeking, fragmented service delivery, inconsistent access to evidence-based care and a lack of standardized outcome indicators capturing functional and social recovery. Innovative approaches included biopsychosocial-spiritual rehabilitation models, peer-led interventions, intra-systemic employment pathways, and symbolic forms of social recognition. In this context, the biopsychosocial-spiritual approach refers to integrative rehabilitation models that extend beyond traditional frameworks by incorporating meaning-making, identity reconstruction, and value-based recovery processes. **Conclusions:** The findings highlight the need to reconceptualize PTSD rehabilitation as a multidimensional, system-level process. In light of the 2023 “Iron Swords” war and the scale of trauma exposure in Israel, the review informs actionable recommendations for developing a coordinated national rehabilitation strategy that integrates clinical care with occupational, community and cultural recovery.

## 1. Introduction

Post-Traumatic Stress Disorder (PTSD) currently constitutes one of the central challenges in the field of mental health for military veterans in Western countries. However, rather than being viewed solely as an individual psychological disorder, PTSD has increasingly been conceptualized as a systemic neuropsychological injury, reflecting complex interactions between biological, psychological, social, and institutional factors. This shift has led to the development of diverse rehabilitation models and treatment approaches, which differ from one another in their theoretical approach, institutional structure, the scope of responsibility borne by the state versus the third sector, and in the methods of evaluating the success of interventions. PTSD represents a substantial burden among military and veteran populations, although prevalence estimates vary across exposure contexts and study populations [1]. This conceptualization reflects the intersection of neurobiological dysregulation, psychological meaning-making, and social–structural determinants such as institutional trust, occupational opportunities, and cultural narratives of trauma. Within this framework, recovery cannot be reduced to symptom remission alone but must be understood as a multidimensional process involving functional reintegration, wellbeing, and long-term participation across life domains. This conceptualization is supported by contemporary research describing PTSD as a multidimensional condition involving neurobiological, psychological, and social determinants [2,3].

The present review examines rehabilitation models in the United States, Canada, the United Kingdom, Germany, Australia, and the Netherlands, analyzing the cultural, organizational, and clinical contexts of each. These countries were selected because they represent OECD systems with established veteran support infrastructures, while also differing in healthcare organization, compensation frameworks, and the balance between military, public, and community-based rehabilitation pathways.

Within this, the guiding principles of each model, barriers to policy implementation at the national level, and challenges of long-term measurement and evaluation are reviewed. The comparison reveals fundamental differences but also points of similarity, mainly regarding the gap between policy declarations and the actual accessibility of effective rehabilitation services.

Accordingly, the aim of this narrative comparative review is to identify shared structural challenges and innovative rehabilitation approaches across Western countries. A further objective is to derive practical implications for Israel.

In light of the “Iron Swords” war and the dramatic rise in the number of citizens and soldiers exposed to prolonged trauma, there is an urgent need to draw practical conclusions relevant to the State of Israel. For this purpose, understanding the systemic dynamics of OECD countries in dealing with military PTSD may provide an essential basis for building an innovative, comprehensive, and effective rehabilitation model in Israel.

Beyond descriptive comparison, this review aims to articulate core rehabilitation principles emerging across Western systems and to translate them into a conceptual framework for national PTSD rehabilitation policy development. In doing so, the paper seeks to shift the discussion from symptom-oriented treatment models toward function-oriented, system-level rehabilitation strategies applicable to Israel and comparable contexts.

## 2. Methods

We conducted a narrative comparative review aimed at mapping and interpreting national-level rehabilitation models for military-related PTSD across selected Western countries. A narrative approach was chosen due to the heterogeneity of the available sources, which included policy documents, governmental reports, institutional publications, and peer-reviewed empirical literature that were not readily amenable to formal meta-analytic synthesis. Rather than estimating pooled effects, the aim was to compare how different national systems conceptualize and organize rehabilitation. This approach is consistent with prior narrative and policy-oriented reviews in complex, multi-level health systems where interventions are not directly comparable.

Peer-reviewed articles and national policy documents published between 2014 and 2023 were identified through searches of PubMed, PsycINFO, and Google Scholar, supplemented by targeted searches of governmental and veterans’ affairs agency websites. Search terms included combinations of “PTSD rehabilitation,” “military veterans,” “national policy,” “peer support,” “Whole Health,” “Operational Stress Injury,” “psychotrauma centers,” and “veteran reintegration”.

Publications were considered eligible if they addressed system-level rehabilitation policies or programs for military veterans with PTSD, including clinical, psychosocial, occupational, community-based, or cultural components. Excluded were studies focused solely on acute trauma response, non-military populations, individual clinical trials without relevance to national rehabilitation structures, and opinion pieces lacking empirical or policy grounding.

The review focused on six countries—the United States, Canada, the United Kingdom, Germany, Australia, and the Netherlands—selected for their comparable OECD contexts and established veteran rehabilitation infrastructures. From each source, we extracted descriptive information regarding: (a) principal rehabilitation services; (b) compensation and entitlement frameworks; (c) employment and occupational integration pathways; (d) community, peer, and cultural anchors; and (e) reported barriers, monitoring practices, and outcome indicators. Although a formal systematic review process was not undertaken, the review followed structured principles of source selection, thematic extraction, and cross-national comparison. Sources were appraised qualitatively on the basis of relevance, methodological transparency, and their contribution to understanding national rehabilitation structures and outcomes.

Findings were synthesized thematically and comparatively across countries, with the aim of identifying shared structural challenges, distinctive national approaches, and transferable principles relevant to policy development in Israel.

## 3. Comparative Findings Across Countries

To enable systematic cross-national comparison, the analysis was structured around five domains: (1) clinical and therapeutic infrastructure; (2) compensation and entitlement frameworks; (3) occupational and functional reintegration pathways; (4) community and cultural integration; and (5) monitoring and outcome evaluation systems.

### 3.1. United States—Multiplicity of Services, Lack of Integration, and Problematic Success Indicators

In the United States, there is a complex system of services for veterans, led by the Department of Veterans Affairs (VA), which includes a wide array of clinics, rehabilitation centers, psychiatric treatment, individual and group psychotherapy, pharmacological treatment, employment programs, legal services, social support, and family services. However, despite this extensive infrastructure, there are significant gaps in service availability, quality monitoring, and the extent to which evidence-based care is implemented. A comprehensive report by the Institute of Medicine identified substantial variability in the delivery of guideline-concordant PTSD care within U.S. veteran systems and highlighted limitations in the systematic measurement of treatment outcomes [1]. Taken together, these findings suggest that fragmentation in the U.S. model is not merely a general impression but a recurrent structural feature, particularly in the coordination between medical, psychosocial, and community-based services.

The models implemented also include innovative approaches such as Whole Health, which conceptualizes veterans as active partners in recovery [4], and programs such as Warrior PATHH, a peer-delivered post-traumatic growth-oriented training model described in the literature as incorporating structured group processes and long-term follow-up [5]. At the same time, while these programs are conceptually influential and operationally important, the empirical evidence regarding their long-term effectiveness remains heterogeneous and still developing.

These findings highlight systemic fragmentation within the U.S. model, particularly in coordination between medical, psychosocial, and community-based programs.

### 3.2. Canada—Systemic Discourse Shift, Peer Support Program, and the OSI Model

Canada chose a cognitive and discursive shift by adopting the term Operational Stress Injury (OSI) within its veteran support framework, reflecting an alternative framing to PTSD that is integrated into national peer-support programs [6]. Services in Canada include a federal rehabilitation program, medical services, psychological treatment, and employment support. The flagship model is OSISS—Operational Stress Injury Social Support—a peer-support program developed by veterans themselves, based on group support addressing moral dilemmas, social isolation, and the gap between combatant identity and post-service identity. Unlike the U.S., Canada operates a structured compensation framework that includes both long-term income support and lump-sum disability awards for eligible veterans [7]. The Canadian case illustrates how diagnostic framing and peer-led support may function not only as service components, but also as mechanisms for reducing stigma and facilitating engagement with care.

The Canadian model demonstrates the impact of terminology and peer-led frameworks on reducing stigma, but structural coordination challenges remain.

### 3.3. United Kingdom—Institutional Medical Model, Few Civilian Players, and Stigma Discourse

The UK operates a national service called Op Courage, part of the public health system (NHS), which provides veterans with access to specialist mental health care and coordinated support pathways [8]. Studies of UK veterans have nevertheless identified barriers to care, including delays in accessing services and stigma-related reluctance to seek treatment [9].

Unlike other trends, in the UK there is a statute of limitations for PTSD claims, usually within seven years from discharge, which creates a particular difficulty for those suffering from late-onset PTSD [10].

These legal constraints uniquely disadvantage veterans with delayed symptom onset, reducing access to compensation and care. This issue is especially significant considering the recognized clinical phenomenon of delayed or late-onset PTSD, in which substantial symptoms may emerge years after the original trauma exposure, often in association with retirement, cumulative stress, aging, or renewed triggers. In contrast, systems such as those in the United States and Canada generally allow claims without similarly rigid temporal limitations, reflecting a broader recognition of the evolving and delayed course that trauma-related disorders may take.

### 3.4. Germany—Intra-Military Rehabilitation, Psychotrauma Centers, and Strict Privacy Regulations

In Germany, PTSD treatment for active-duty personnel is delivered primarily within the Bundeswehr medical system, including specialized military psychiatric and psychotrauma services [11,12]. Care is embedded within military hospitals and structured psychiatric rehabilitation pathways. Rehabilitation planning may involve adapted service roles or structured transition processes within the defense framework. Published analyses describe a clinically centered model anchored in institutional psychiatric care within the armed forces [11,12].

This model reflects a centralized institutional framework in which care is delivered predominantly within military medical structures. Such an arrangement may support continuity of care, preserve military identity, and facilitate adapted service roles; however, it may also limit integration with civilian rehabilitation pathways and reduce the visibility of long-term social reintegration outcomes.

### 3.5. Netherlands—Institutional–Societal Approach, Symbolic Reinforcement, and Veteran Support

The Netherlands adopts a broad approach to rehabilitation that also includes symbolic components—such as a national Veterans’ Day, integrating veterans into state ceremonies, and granting public honor to post-trauma as an inseparable part of service. Rehabilitation is sometimes carried out in cooperation with community cooperatives, emphasizing social-community experience as a component of recovery [13].

Symbolic recognition occupies a visible institutional role within the Dutch veteran framework, differentiating it in emphasis from systems that prioritize primarily clinical service delivery. The Dutch case may therefore be understood as a hybrid model in which symbolic recognition is not merely ceremonial, but functions as part of a broader rehabilitative ecology linking public acknowledgment, identity reconstruction, and community reintegration.

### 3.6. Australia—Group Resilience and Broad Cultural Context

In Australia, the Department of Veterans’ Affairs (DVA) delivers a range of mental health and rehabilitation services that include individual treatment, family support initiatives, and community-based programs [14]. Policy frameworks emphasize wellbeing, recovery, and social participation within veteran support services. Compared with some of the other systems reviewed, the Australian model appears to place relatively greater emphasis on family-inclusive and community-based rehabilitation, thereby extending recovery beyond the clinic into broader social participation. This emphasis suggests a broader rehabilitation logic in which family support and community participation are treated not as secondary adjuncts, but as integral components of recovery. To facilitate cross-national comparison, the key structural features identified across countries are summarized in Table 1.

Although direct quantitative comparison across countries is limited by heterogeneity in reporting and outcome measurement, a broader pattern emerges in which systems with more integrated service structures tend to offer more developed reintegration pathways, even if they continue to face fragmentation and monitoring limitations.

## 4. Discussion

### 4.1. Cross-Country Challenges in Military PTSD Treatment and Rehabilitation

#### 4.1.1. Stigma as a Systemic and Personal Barrier to Seeking Care

One of the central challenges recurring across almost all reviewed countries is stigma, which continues to deter many veterans from seeking professional mental health care [15,16]. This observation is consistent with prior empirical work showing that stigma, anticipated career consequences, and internalized perceptions of weakness remain among the most persistent barriers to help-seeking in military and veteran populations. This interpretation is consistent with review literature indicating that stigma remains one of the most significant barriers to care in military populations [17,18].

Stigma operates on two levels: first—the fear of social labeling as “weakness” or “mental failure,” and second—the concern about harm to future employment potential, especially among veterans of combat units or security systems, where continuity of service or suitability for positions of responsibility is required.

Research among combat veterans has consistently demonstrated that stigma-related concerns and perceived career consequences significantly reduce help-seeking behavior and delay treatment engagement, particularly among combat-exposed personnel embedded in military cultures emphasizing toughness, independence, and self-control [15,16,19]. In countries such as the UK, Germany, and Israel, where seeking treatment may be perceived as “undermining combat morale,” treatment-seeking rates are significantly lower, especially among active combatants. This dual-layered stigma creates both interpersonal and institutional barriers that substantially delay treatment entry.

#### 4.1.2. Measurement and Monitoring Gaps: What Constitutes Success in PTSD Treatment?

Another structural issue is the lack of agreed success indicators and effective monitoring systems. Most developed countries offer a wide range of treatments—psychological, pharmacological, rehabilitative, and occupational, yet few models include structured, longitudinal outcome monitoring capable of assessing functional, social, and occupational recovery trajectories.

A prominent example can be found in the U.S., where a national review determined that despite enormous investment, only a small fraction of diagnosed individuals receive evidence-based care, and there is no uniform definition of functional improvement or recovery [1], limiting cross-national comparability and policy learning. This makes it difficult to determine whether treatment is successful and whether it promotes social and occupational integration. One potential way of addressing this gap is the incorporation of standardized functional outcome measures alongside symptom measures, for example tools assessing social adaptation, occupational participation, and quality of life. Such measures could help bridge the divide between clinical improvement and real-world rehabilitation outcomes. Related research emphasizes that symptom reduction alone is insufficient to capture meaningful recovery, and that functional outcomes such as occupational performance and community reintegration should be considered core rehabilitation indicators [20,21].

#### 4.1.3. Cultural and Systemic Gaps in Treatment

Most systems rely on the medical–individual treatment paradigm—namely, individual treatment focusing on the person through psychotherapy (primarily CBT) and pharmacological care. While this approach has research-based advantages, it often overlooks cultural, community, and family contexts that are an essential part of the veteran’s identity. In WEIRD (Western, Educated, Industrialized, Rich, Democratic) countries, whose psychological research traditions have been critiqued for cultural narrowness, treatment delivery often remains predominantly clinically centered rather than socially embedded [22]. The missing emphasis on systemic and social treatment risks overlooking the complexity of the injury and the links between PTSD, isolation and loss of meaning. Family systems are also a critical yet often under-integrated component of rehabilitation. In practice, long-term recovery is shaped not only by the individual veteran’s symptoms, but also by relational dynamics, caregiving burdens, and the family’s role in supporting or constraining reintegration.

#### 4.1.4. Late-Onset PTSD and Regulatory Implications

Late-onset PTSD refers to the emergence or marked worsening of trauma-related symptoms long after service completion, sometimes years or decades later.

This phenomenon has been described among veterans who appear functionally stable for extended periods but experience symptom emergence later in life, sometimes following major life transitions or renewed stress exposure.

While in the U.S. and Canada it is possible to file assistance requests at any stage, in the UK claims under the Armed Forces Compensation Scheme must generally be submitted within seven years of the earliest qualifying date, which may include discharge [10]. This regulatory barrier disproportionately disadvantages veterans with delayed symptom onset and restricts equitable access to care. This issue is not sufficiently addressed in many countries, including Israel.

#### 4.1.5. Gender-Specific Considerations

Female veterans may face distinct patterns of trauma exposure, including higher rates of interpersonal trauma and military sexual trauma, as well as different clinical trajectories and help-seeking patterns [23]. As female soldiers take a larger proportion of military and combat roles, a more comprehensive rehabilitation policy framework should therefore include gender-sensitive service design and outcome evaluation.

### 4.2. Innovative Models—New Approaches to Post-Traumatic Rehabilitation

#### 4.2.1. The Biopsychosocial–Spiritual Approach

Instead of focusing only on psychological and medical aspects, several groundbreaking programs—such as Whole Health in the U.S.—are developing holistic rehabilitation models. This approach integrates the body, mind, social support, and the spiritual or value dimension of the individual in the recovery process [4]. Conceptually, this approach extends traditional models by incorporating meaning-making and identity reconstruction processes, which have been shown to play a central role in psychological adaptation to trauma [24]. The focus is on the question “What am I healing for?” rather than only “What am I suffering from?” This process includes deep conceptual change, strengthening the individual’s involvement in setting treatment goals, and sometimes the use of arts, bodywork, and community as rehabilitation supporters. This holistic framing positions recovery not only as symptom reduction but as restoration of meaning, agency, and coherence in the veteran’s life. Recent work has further supported this orientation by suggesting that interventions targeting thought management, agency, and cognitive–emotional regulation may contribute not only to subjective wellbeing, but also to productivity and leadership functioning, thereby reinforcing the relevance of meaning-centered rehabilitation models [25].

#### 4.2.2. Peer-Based Rehabilitation

Programs such as OSISS in Canada and Warrior PATHH in the United States utilize peer-based models in which veterans with lived experience provide structured support to others in recovery [5,6]. Such models emphasize identification, shared experience, and reduction in isolation as core components of rehabilitation. In Israel, no formal similar model has yet been institutionalized aside from local NGO initiatives. Peer-led models provide a unique therapeutic mechanism through shared lived experience, which enhances trust, reduces stigma, and increases sustained engagement in care.

#### 4.2.3. Intra-Systemic Employment Integration

In Germany and in some U.S. cases, there is an option for continued employment within the defense system or alongside it, even for traumatized veterans. This integration allows for preservation of a sense of meaning, belonging, and identity, and serves as an important bridge to functional recovery, adapting employment conditions to the veteran’s psychological state. Such employment pathways illustrate how institutional reintegration can serve simultaneously as treatment, rehabilitation, and identity stabilization. Importantly, successful occupational reintegration is shaped not only by symptom severity, but also by broader predictors such as stress resilience, interpersonal functioning, and educational background. Integrating such predictors into rehabilitation planning may improve the precision of return-to-work support and long-term vocational outcomes [26]. This is consistent with findings showing that improvements in PTSD symptoms do not necessarily translate into full occupational recovery, highlighting the need to assess work functioning as an independent outcome domain [21].

#### 4.2.4. Positive Cultural Framing and Social Recognition

The framing of the injury plays a central role in its impact on both the individual and society. In Canada, for example, the term OSI—Operational Stress Injury—was adopted as an alternative to “Post-Traumatic Stress Disorder,” emphasizing that it is an injury, not a personality failure or chronic disease [6]. In the Netherlands, national ceremonies, social recognition, and positive language that speaks of “service and pride,” rather than fracture, are prominent. Conversely, in the UK, many veterans report alienation, shame, and a perception of “fragility,” which hinders seeking help [9]. Cultural framing thus functions as a therapeutic variable in itself, influencing identity reconstruction, help-seeking behavior, and societal reintegration.

#### 4.2.5. The Need for Effective National Monitoring and Measurement

Across all models, the need arises to establish an effective, national, and uniform monitoring system that allows for understanding of treatment and rehabilitation effectiveness over time—not only by clinical measures, but also by social, occupational, and family functioning. Such systems require systemic investment in technology, data collection, and transparency. Without standardized national indicators, it becomes difficult to allocate resources efficiently, compare program effectiveness, or design evidence-based policy reforms. In practical terms, such systems may include digital platforms for longitudinal symptom tracking, telehealth services for geographically or socially isolated veterans, and mobile tools that support self-monitoring and early intervention between formal treatment contacts.

### 4.3. Limitations

This narrative review has several limitations. The analysis relied on publicly available literature and policy documents and did not include formal quality appraisal or quantitative synthesis. Country selection was purposive and policy-oriented rather than exhaustive. Accordingly, findings should be interpreted as conceptual and system-level insights rather than epidemiological comparisons. At the same time, the narrative design enabled the integration of policy documents, institutional frameworks, and empirical findings into a comparative system-level synthesis that may have been more difficult to capture through a narrowly clinical review methodology alone.

#### Implications and Recommendations for Israel

International evidence increasingly suggests that functional recovery and long-term participation outcomes constitute the primary determinants of successful PTSD rehabilitation. Accordingly, the central policy question is not only how to reduce symptoms, but how to restore participation, belonging, employability, and continuity of life over time. Taken together, these cross-national findings suggest that effective PTSD rehabilitation depends less on individual treatment modalities and more on system-level organization and functional recovery orientation. Following the events of October 2023 and the unprecedented scope of trauma exposure—among combatants, civilians, reservists, medical teams, and families—it is clear that Israel requires a large-scale national program for treating PTSD. In light of the high proportion of military veterans in the population, the multiplicity of trauma exposures, and the ongoing strain and fragmentation within the public health system, there is an urgent need to adopt a systemic, multidisciplinary, gender sensitive and continuous approach, integrating advanced medical care, community support, occupational guidance, and cultural redesign of public discourse. From the cross-country review it emerges that advanced countries have in recent years adopted comprehensive models including not only clinical response to PTSD, but also social, occupational, and narrative rehabilitation.

These approaches, including the U.S. Whole Health program [4], the Canadian OSISS network [6], Germany’s institutionally embedded psychiatric care model [11,12], and the Dutch emphasis on symbolic recognition [13], underscore the importance of community, peer support, meaningful experience, and public acknowledgment within veterans’ rehabilitation frameworks.

These models highlight gaps in Israel’s current approach, which remains primarily clinically oriented and lacks coordinated community-based and peer-led components.

The lessons learned from these countries provide a foundation for deriving practical principles relevant to Israel, including: establishing a national center for PTSD treatment and rehabilitation, developing a network of “rehabilitation ambassadors” among veterans, integration into designated public-sector employment pathways as part of the therapeutic process, providing treatment access for late-onset PTSD [10], formulating national success indicators including medical, functional, and social metrics, and finally, a fundamental shift in public discourse—from framing it as a personal “fracture” to broader societal narratives of resilience, coping, and collective responsibility.

These insights can be translated into concrete policy directions at the national level. The principal policy recommendations emerging from the cross-country analysis are operationalized and summarized in Table 2.

## 5. Conclusions

This review suggests that the central challenge in PTSD rehabilitation is not the absence of effective treatments, but the way these treatments are embedded within broader systems of care. Across the countries examined, similar tensions repeatedly emerge-between clinical knowledge and real-world accessibility, between symptom reduction and functional recovery, and between individual treatment and the wider social context in which recovery unfolds. What becomes apparent is that rehabilitation succeeds not when services are expanded in isolation, but when they are coherently integrated. Models that combine clinical care with peer support, occupational pathways, and meaningful social participation appear better positioned to support sustained recovery over time, particularly in populations exposed to prolonged or repeated trauma. In the Israeli context, these insights carry immediate relevance. The scale and complexity of trauma exposure following the 2023 “Iron Swords” war highlight the limitations of a predominantly clinic-centered response. Addressing this challenge will require not only additional resources, but a conceptual shift toward a coordinated, system-level approach that links treatment, rehabilitation, and social reintegration. Ultimately, the findings point toward a broader redefinition of recovery itself—from the alleviation of symptoms to the restoration of function, meaning, and continuity of life. Future work should therefore focus on developing integrated national frameworks and on establishing outcome measures that capture not only clinical change, but real-world recovery across domains of living.

## Figures and Tables

**Table 1:** Comparative Rehabilitation Models for Military PTSD.

Country	Main Medical Services	Financial Compensation	Employment Integration	Community/Cultural Anchor	Main Barriers
United States	VA, PE/CPT, Whole Health [4]	Monthly pension	Service within VA	Warrior PATHH approach	Lack of integration; poor success indicators
Canada	OSI Clinics; integrative care	Pension/grant	OSISS programs	Non-stigmatic public discourse	Regulatory inconsistency
United Kingdom	NHS (Op Courage); psychotherapy	One-time grant/ongoing allowance (AFCS)	NGO complementary	Stigma; limited availability	Time limit; regional variation
Germany	Psychotrauma centers; military rehabilitation	Rehabilitation package	Continued adapted service	Closed regulation; limited community role	Lack of epidemiological indicators
Netherlands	Holistic treatment	Tailored compensation	Cooperative integration	National ceremonies; symbolic honor	Lack of uniform rehab standard
Australia	Group resilience; family therapy	Inclusive compensation	Community rehabilitation	Active support communities	Center–periphery gap

**Table 2:** Cross-Country Models and Recommendations for Israel.

Action Area	Practical Recommendation	Inspiration from Cross-Country Models
National Treatment Center	Establishment of a national PTSD center integrating medical, psychosocial, and community care in one place	Whole Health (U.S.)
Peer Support	Development of ‘rehabilitation ambassadors’—recovering veterans serving as mentors	OSISS (Canada); Warrior PATHH (U.S.)
Public Occupational Rehab	Creation of adapted jobs as part of rehabilitation, not only placement assistance	Bundeswehr (Germany); VA (U.S.)
Late-Onset PTSD	Providing treatment access also for Late-Onset PTSD	Lessons from UK model
National Success Indicators	Formulating medical, occupational, and social indicators for assessing rehabilitation success	VAC (Canada); DVA (Australia)
Public Discourse Change	Transition to a discourse of ‘healing,’ ‘coping,’ and ‘moral pride’ around military trauma	OSI (Canada); national ceremonies in the Netherlands

## Data Availability

No new data were created or analyzed in this study. Data sharing is not applicable to this article.
